# Improved Production of Industrially Important Essential Oils Through Elicitation in the Adventitious Roots of *Artemisia amygdalina*

**DOI:** 10.3390/plants8100430

**Published:** 2019-10-20

**Authors:** Faqeer Taj, Mubarak Ali Khan, Huma Ali, Raham Sher Khan

**Affiliations:** Department of Biotechnology, Faculty of Chemical and Life Sciences, Abdul Wali Khan University Mardan (AWKUM), Mardan 23390, Pakistan

**Keywords:** Artemisia, Adventitious roots, elicitation, osmotic stress, antioxidant, volatiles, essential oils

## Abstract

The limited production of bioactive essential oils in natural plants does not meet the increasing worldwide market demand. Plant cell culture technology can be used for the higher production of industrially important essential oils. In the present study, a suitable method for production of essential oils was developed through establishment and elicitation of adventitious roots (AR) in a medicinally important plant *Artemisia amygdalina* D. The results indicated that leaf explants cultured on solid Murashige and Skoog (MS) media supplemented with 1.0 mg/L α- naphthalene acetic acid (NAA) and 4% sucrose instigated the higher AR induction frequency (90  ±  4.25) and maximum AR biomass (fresh biomass: 17.7 g/L). Furthermore, in the AR when transiently elicited with different elicitors for different time periods, methyl jasmonate (Me-J: 0.5 mg/L) resulted in the higher production of total phenolic content (TPC: 3.6 mg), total flavonoid content (TFC: 2.3 mg) and phenylalanine ammonia-lyase (PAL: 4.8 U/g×FW) activity, respectively. Nonetheless, considerable levels of the major bioactive compounds such as α-thujene (6.8%), α-pinene (8.3%), 1,8-cineole (16.2%), camphor (8.4%) and verbenole (10.2%) were recorded in the Me-J treated AR. Thus, a feasible protocol for production of essential oils through AR in *A. amygdalina* was established, which can be exploited for commercial production of the industrially important terpenes.

## 1. Introduction

Herbal medicines are getting resurgence for their worldwide recognition in the treatment of many diseases, especially in the era of antibiotic resistance, where many commercially available synthetic antibiotics are becoming impotent against several human diseases [1,2]. The therapeutic potential of these herbal medicines is by virtue of the presence of important natural products also called secondary metabolites. Medicinal plants act as a rich repository of a variety of the bioactive secondary metabolites and provide the raw material to pharmaceutical industries for the formulation of herbal products [3]. The genus *Artemisia* of family Asteraceae has 500 species worldwide, majority of the plant species are medicinally and economically important [4]. Amongst the medicinal plants from this genus, *Artemisia amygdalina* D. is one of the vital plants, reported for its diverse pharmacological and ethno medicinal uses by people from different communities. It is found across Asia, Europe and North America. In Pakistan, it is found in the subalpine region of Khyber Pakhtunkhwa and Kashmir, where locally it is known as Veer Thethven [5,6,7]. *A. amygdalina* is a 1.5 m tall, stout stemmed leafy, aromatic perennial herb with simple serrate leaves that are hoary tomentose beneath and glabrous green above, flower heads of 2–3 mm, bearing short axillary racemes [8]. The plant has been used locally since old ages for the treatment of many diseases, including epilepsy, piles, nervous disorders, cough, cold, fever and pain [9,10]. The plant extracts have shown promising results in many biological activities, including anti-oxidant, anti-bacterial, anti-inflammatory, anti-microbial and immunomodulatory activities [7,10,11,12]. The major bioactive compounds reported in *A. amygdalina* are the terpenes, p-cymene, 1,8-cineole and artemisinin [13]. However, other medicinally potent secondary metabolites, such as flavonoids, tri-terpenoids, alkaloids, saponins, cardiac glycosides, and steroids, are also found in different plant parts extracted with either aqueous, chloroform, ethyl acetate, ethanol or methanol solvents [10,11]. Due to their higher medicinal activity, *A. amygdalina* plants in their natural habitat have been over-harvested and exploited, not only by the local people but also by different pharmaceutical industries for making different herbal products. Furthermore, deforestation has drastically eroded this plant species in its natural reservoirs and it has been listed in the critically endangered plant species [5]. Conventional propagation in *A. amygdalina* through seeds is limited due to the higher mortality rate of seedlings in early stages of growth. Biotechnological methods, especially plant cell culture technology, has provided suitable platforms for the production of healthy plant material and useful secondary metabolites year-round in any season under aseptic growth conditions in a limited space and shorter time [2,14,15]. Adventitious roots (AR) culture technology has been proven as an efficient cell culture technology for quality production of biomass and health-promoting metabolites and is also called a metabolites biosynthetic factory [16,17]. Although induction and growth of AR is a complex process, several factors, such as nutritional status of the culture media, type and concentration of phytohormones and in vitro growth conditions, are directly linked to AR growth and proliferation [18]. Elicitation is one of the most successful strategies used in vitro for enhancement of production of plant secondary metabolites in a lot of medicinal plants, in order to meet the higher industrial demands for making pharmaceutical products and drugs [2,19]. It is an abstinent method and has been recognized as a propitious technique for the production of the metabolites in many plants species, where applications of synthetic biology, metabolic engineering or genetic transformation methods are difficult to implement due to lack of proper genetic information about the specific metabolite biosynthetic pathway [20,21]. Several types of elicitors for instance methyl jasmonate (Me-J), melatonin and phenylacetic acid, etc. are routinely employed in vitro for the enhancement of different classes of bioactive secondary metabolites in medicinal plants through AR culture technology [19,22]. The present study was therefore aimed to optimize a feasible protocol for AR induction, biomass formation and biosynthesis of important medicinal compounds in *A. amygdalina.* Furthermore, notable elicitors such as Me-J, melatonin and PAA were applied in vitro to enhance the secondary metabolite profiles and production of essential oils in AR.

## 2. Materials and Methods

### 2.1. Plant Material, Sterilization and Explant Preparation

Seeds of *Artemisia amygdalina* were obtained from the seed bank at the plant cell culture laboratory, Abdul Wali Khan University Mardan, Khyber Pakhtunkhwa, Pakistan. Viable and healthy seeds were selected for germination. Surface sterilization of seeds was employed by washing in tap water for 25 min, followed by treatment in 70% ethanol for 5 min and with 0.1% (*w*/*v*) solution of mercuric chloride (HgCl_2_) for 5 min, and finally, the seeds were washed with sterile distilled water five times. Sterilized seeds were placed on filter paper to dry and were subsequently cultured on Murashige and Skoog (MS) [23] medium, containing 3.0% sucrose and 0.8% agar. Conditions of growth room were 16 h light and 8 h dark photoperiod with light intensity of ~40 μM m^−2^ sec^−1^, 25 ± 1 °C temperature and 70% relative humidity. Young leaf sections (~1.0 cm) were collected from 50 day-old germinated plants and were used for establishment of adventitious roots in the subsequent experiments.

### 2.2. Establishment of Adventitious Roots (AR) Cultures on Solid Media

Leaf explants were surface sterilized before inoculation in the culture growth media. Briefly, leaf segments were first treated with sterile distilled water for 5 min, then treated with 0.1% (*w*/*v*) HgCl_2_ for 5 min and finally rinsed with sterile distilled water three times. For optimization of AR cultures, leaf explants were cultured on MS solid media, supplemented with different auxins such as indole-3-butyric acid (IBA), α-Naphthalene acetic acid (NAA) or indole-3- acetic acid (IAA) at different levels (0.5, 1.0 or 1.5 mg/L). The media also contained 3.0% sucrose and was solidified with 0.8% agar. The pH of media was maintained at 5.8, following autoclaving for sterilization at 121 °C (105 kPa) for 18 min. For control treatment MS, media containing no auxin was used. All the culture flasks were placed under growth room conditions of 16 h light and 8 h dark photoperiod with light intensity of ~40 μM m^−2^ sec^−1^, 25 ± 1 °C temperature and 70% relative humidity. After a four-week culture period, the data were recorded as percentage of AR induction (responding explants/ total non-contaminated explants into 100), days to root initiation, number of AR per explant (mean), AR length (cm) and biomass (fresh biomass: g/L).

In the second set of experiments, the effects of sucrose at different levels were estimated on AR induction and growth. Leaf explants were cultured on the MS solid media containing an optimal level of PGR (1.0 mg/L NAA) and augmented with the different concentrations (1–6%) of sucrose. The media composition and the growth conditions were maintained the same as used in the initial AR optimization experiments. Media supplemented with 3% sucrose was considered as control treatment. After a four-week culture period, the data were recorded as percentage of AR induction (responding explants/ total non-contaminated explants into 100), days to root initiation, number of AR per explant (mean), AR length (cm) and biomass (fresh biomass: g/L).

### 2.3. Transient Elicitation of AR and Cultivation in Shake Flask Liquid Media

AR grown over solid media containing 1.0 mg/l NAA and 3% sucrose were taken and pretreated with different elicitors for different time periods (transient elicitation). Elicitors used were melatonin (Mel), methyl jasmonate (Me-J) and phenylacetic acid (PAA). The AR were treated with each elicitor at concentration of 0.5, 1.0 or 1.5 mg/L for 30, 60 or 90 min, respectively, and after elicitor treatments, approximately 2 g/L of AR were transferred into liquid MS media containing 1.0 mg/L NAA and 3% sucrose, following incubation on rotary shaker at 110  rpm for a 30-day culture period. The rotary shaker conditions were adjusted at 16 h light and 8 h dark photoperiod with light intensity of ~40 μM m^−2^ sec^−1^, 25 ± 1 °C temperature and 70% relative humidity. The data were recorded as proliferation of AR (%) and AR fresh biomass (g/L) in response to each elicitor treatment.

### 2.4. Phytochemical Analysis for the Assessment of Effects of Elicitation on the Production of Secondary Metabolites in the In Vitro Raised AR

Different samples of Adventitious roots (AR) established in vitro in the present study were selected on the basis of maximum growth in the respective culture treatments and were subjected to different assays for phytochemical analysis. AR samples were annotated as NAA-AR (AR grown over solid MS media containing 3% sucrose and 1.0 mg/L NAA), Sucrose-AR (AR grown over solid MS media in the presence of 4% sucrose and 1.0 mg/L NAA), Mel-AR (AR pretreated with melatonin following culturing in liquid MS media containing 3% sucrose and 1.0 mg/L NAA), MeJ-AR (AR pretreated with methyl jasmonate following culturing in liquid MS media containing 3% sucrose and 1.0 mg/L NAA) and PAA (AR pretreated with phenylacetic acid following culturing in liquid MS media containing 3% sucrose and 1.0 mg/L NAA). All the AR samples were compared with the in vitro raised plants (control plant sample) for the estimation of secondary metabolite profiles.

For extract preparation, all the selected samples were dried in a hot air oven at 50 °C for two days and were powdered using mortar and pestle. Each sample weighing dried two grams were taken separately in 50 mL and was mixed with 10 mL of 50% methanol (HPLC grade, Merck, Germany), placed on a shaker (24 rpm; 24 h; room temperature), sonicated (30 min), vortexed (5 min) and sonicated again (15 min). It was then centrifuged (6500 rpm; 10 min) and the supernatant was syringe filtered and transferred to Eppendorf tubes. The plant extract was diluted to a final concentration of 10 mg/mL for a uniform analysis. The extract solution was stored at 4 °C until used.

#### 2.4.1. Determination of Total Phenolic Content (TPC)

The Folin-Ciocalteu assay was employed for the estimation of TPC in the selected samples, according to the method of Velioglu et al. [24]. The assay was expedited in 96 wells plate by adding 20 μL (10 mg/mL) of the test sample to each well followed by addition of 90 μL of Folin-Ciocalteu reagent (10×) with a ratio of 1:9 and incubated for 5 min at 25 °C. After incubation, 90 μL of 6% stock solution of Na_2_CO_3_ (6 g/mL) was added to all the wells and mixed properly. DMSO (20 μL) was taken as a negative control, while 20 μL (4 mg/mL methanol) of Gallic acid was used as a positive control. The plate was incubated for 90 min at 25 °C. OD was measured at 630 nm using microplate reader (ELX 800, BIOTEK).The results were expressed as gallic acid equivalent (GAE) mg/g DW of extracts.

#### 2.4.2. Determination of Total Flavonoid Content (TFC)

Total flavonoid content was determined by using the established protocol of Chang et al. [25]. In a 96 well plate, 20 μL of each methanolic extract was added with 10 μL of both potassium acetate (98.15 g/L H_2_O) and aluminum chloride (10% *w*/*v*). The final volume of the reaction mixture was raised by addition of 160 μL of distilled water and was adjusted at 200 μL. Following incubation in dark condition for approximately 30 min, the absorbance of reaction mixture was taken at 450 nm on (ELX 800, BIOTEK) microplate reader. Quercetin was used as positive and methanol as negative control. The results were expressed as mg quercetin equivalent (QAE) mg/g DW of extracts.

#### 2.4.3. Phenylalanine Ammonia Lyase (PAL) Activity

The method adopted by Khan et al. [26] was utilized for the determination of PAL activity. Briefly, 80–100 mg FW of each plant sample was homogenized with ice-cold potassium borate buffer (100 mM, pH 8.8) and mercaptoethanol (2 mM), followed by centrifugation for 10 min at 12,000 rpm at 4 °C. Following centrifugation, the residual pellet was discarded and the supernatant was used in the assay. The reaction mixture consisted of 1 mL potassium borate buffer (100 mM), 0.5 mL of 4 mM^−1^ phenylalanine and 0.5 mL of sample at pH 8.8. The reaction mixture was incubated at 30 °C and further 0.2 mL HCl was added to stop the reaction. Once the reaction was stopped, after 30 min, absorbance was recorded at 290 nm. The quantity of synthesized trans-cinnamic acid was calculated using its 9630 M^−1^ cm^−1^ extinction coefficient. The increase in absorption was reported depending on the quantity of the product produced. In particular, one unit of PAL operation (U) was described as the quantity of absorbance at 0.01 variation. The assay was repeated thrice and the mean value of the ΔA270 nm/min was obtained using the maximum linear rate for both the test sample and blank.

#### 2.4.4. DPPH Free Radical Scavenging Assay

DPPH (α, α-diphenyl-β-picryl hydrazyl) based assay was employed for the determination of antioxidant potential in the selected plant samples according to the method of Abbasi et al. [27]. In a reaction mixture, 20 μL of each methanolic extract was added with 180 μL of DPPH solution (4.8 mg/50 mL), following incubation in dark condition for approximately 60 min. Furthermore, the reaction mixtures containing 180 μL of DPPH in combination with 20 μL DMSO or ascorbic acid were used as negative and positive controls, respectively. The absorbance of each reaction mixture was measured at a wavelength of 517 nm.

The free-radical scavenging activity of the sample towards DPPH radical was calculated using the equation:

Antioxidant activity (%inhibition) = (1 − (Abs sample/Abs blank)) × 100

### 2.5. Quantitative Analysis for the Assessment of Effects of Elicitation on the Production of Essential Oils in the In Vitro Raised AR Using Gas Chromatography-Mass Spectrometry (GC–MS)

The selected plant samples were subjected to hydro-distillation in a Clevenger type apparatus for extraction of essential oils. For this purpose, fresh biomass (∼15 g) from the cultures of each sample was added with 300 mL of distilled water in a round-bottom flask. After three hours of distillation, anhydrous sodium sulfate was added to the mixture to initiate the drying of volatile compounds. For this purpose, anhydrous sodium sulfate was used as desiccation agent. Essential oil in each plant sample was calculated on the basis of accumulation of dry matter as mL/100 g × dry matter. The extracted samples were kept in air tight vials under dark at 4 °C until further processing. GC-MS apparatus was used for the analysis of volatile organic compounds (VOCs) in the selected samples. A dimethylpolysiloxane-coated capillary column (60 m × 0.25 mm; DBI) was attached to the Shimadzu GC–MS-QP 2020 system. The n-hexane extract (0.2 μL) was injected to the apparatus in split ratio (1:5) with helium as a carrier gas at the rate of 1 mL/min. The mass spectrometer (MS) was configured with different operating conditions such as injection port temperature at 280 °C, column temperature at 50 °C for 2 min followed by an increase to 300 °C at a rate of 3 °C/min, a detector with a temperature of 310 °C, with an ion source 280 °C, ionization energy of 70 eV, solvent delay at 8.0 min, EV voltage of 3000 V, scan speed with 2000 us^−1^ and mass scan range at 30–600 a.m.u. Finally, the mass spectrum profiles of the VOCs obtained for each plant sample were compared with spectra of several standard reference compounds in the plantcyc library (http://pmn.plantcyc.org/). Comparison of the retention indices (RI) of the VOCs in the mass spectral chromatograms of the samples with standards selectively identified the different metabolites in each sample [28,29].

### 2.6. Statistical Analysis

The data were statistically analyzed by one way analysis of variance (ANOVA) test using the statistical software SPSS 16.0 and mean values in each data set were compared using Duncan’s multiple range test procedure (*P* = 0.05).

## 3. Results and Discussion

### 3.1. Effects of Auxins on Induction and Growth of Adventitious Rooting in Explants

The primarily objectives of the current research work were to optimize a feasible protocol for the establishment of adventitious roots (AR) and production of bioactive metabolites in the medicinally important and critically endangered plant *Artemisia amygdalina.* In the initial experiments, leaf explants taken from the in vitro grown *A. amygdalina* plants were cultured on MS basal media, supplemented with different auxins and (3%) sucrose and incubated for a culture period of four weeks. The results indicated that among all the auxins tested in vitro, the highest growth parameters (% AR induction: 87 ± 4.25, number of AR per explant: 6.8, length: 3.2 cm and fresh biomass: 15.7 g/L) were recorded in the explants cultured in the presence of 1.0 mg/L NAA (Table 1). It is a common pattern that cytokinins, preferably benzyl aminopurine (BA), promotes shoot organogenesis in explants. Likewise auxins, notably NAA usually results in root regeneration during in vitro cultures [30,31]. In several medicinal plants, NAA has been reported for its highest activity in AR formation [17]. Within the different auxins, NAA has the potential to interact efficiently with the plant cell during in vitro growth due to the fact that plant cell can uptake it very rapidly from the growth media, for instance when compared with IBA and IAA, the rate of uptake by plant cell in tobacco callus was six times faster for NAA [21]. *A. amygdalina* has not been previously exploited for the establishment of AR cultures; however, many other *Artemisia* species have been explored in vitro for induction and biomass formation of AR. In a recent report, NAA at 1.0 mg/L resulted in the highest AR induction frequency (57%) and growth parameters (mean number of AR per explant: 16.6 and AR fresh biomass: 25.2 g/L) in the leaf explants of *A. scoparia* grown over solid MS media [32]. Moderate morphogenetic potential in explants for AR induction and biomass formation was observed for IBA, wherein the highest growth attributes in terms of AR induction frequency (62%), mean number of roots (2.6), root length (1.15 cm) and fresh biomass (9.5 g/L) were observed at 1.0 mg/L IBA (Table 1). In a similar study, higher AR growth parameters (number of AR: 10.8 and AR length: 13.9 cm) were recorded in leaf explants of *Artemisia vulgaris* cultured on MS solid media supplemented with1 4.9 μM IBA in combination with 1.4 μM IAA [33].

**Figure 1:**
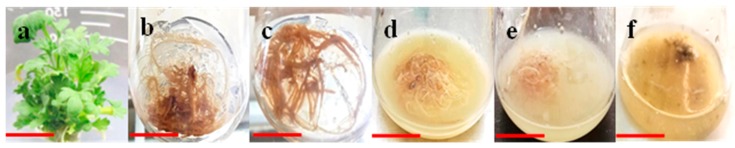
Establishment of in vitro adventitious roots (AR) in *A. amygdalina*. (**a**) In vitro raised plant as a source of aseptic explants, (**b**) AR established in response to 1.0 mg/L NAA and 3% sucrose, (**c**) AR established in response to 1.0 mg/L NAA and 4% sucrose, (**d**–**f**) AR suspension established in response to pretreatment with PAA, Mel and Me-J, respectively.

In another study, the highest AR induction frequency (72.4 ± 9.3%) and biomass production were recorded in leaf explants of *L. pumila,* at 5 mg/L of IBA [34]. In this study, direct AR formation was observed in explants in response to all the auxins treatments. AR formation was visually observed at the cut ends of explants after four days of explants incubation in case of NAA and five days incubation in presence of IBA (Table 1; Figure 1). Both NAA and IBA showed lower growth response when employed in vitro in explants at higher doses (1.5 mg/L). Surprisingly, similar to the control treatment, where explants were cultured on MS0 media (media containing no auxin), no AR induction was observed at IAA tested at all levels in the present study (Table 1). However, it was noticed that IAA supplementation in the culture media resulted in callus formation in explants (data not shown).

Contrarily, IAA addition to MS media at 3 mg/L, resulted in the highest AR induction frequency (88.3%) and biomass production (0.060 g DW) in leaf explants of *Orthosiphon stamineus* [35]. Auxins are considered important for their potential role in influencing the plant cell through its passage at the cut end of explants for induction of AR. This passage of exogenous auxin inside plant cell occurs on an anatomical basis, triggers the indigenous auxin transport movement through distinct physiological mechanisms such as pH trapping and influx carriers [36]. All these interactive events result in the differentiation and induction of AR in explants [16,37].

In the present study, the rate of necrosis and contamination in the explants forming AR was negligible (≤5%; data not shown). This is because the leaf explants used for establishment of AR were taken from the in vitro raised plantlets of *A. amygdalina*, which prevented the in vitro contamination. Moreover, the stringent sterilization protocol that we have developed for the surface sterilization of explants in a variety of medicinal plants those we have cultivated in vitro in our research laboratory also promoted the aseptic AR growth in present study. Contamination during in vitro cultures is troublesome and can be a direct threat to the plant cellular growth, resources and time in optimization of any micro propagation protocol for any medicinally important plants. The source of explants is a critical factor in controlling any sort of in vitro contamination in culture flasks. Explants taken from the plant germplasm raised in vitro under aseptic growth conditions can reduce significantly the level of contamination during in vitro plant cell growth as compared to the explants taken from the wild grown plants [38,39].

### 3.2. Effects of Varying Levels of Sucrose on Induction and Growth of Adventitious Rooting in Explants

Sucrose is one of the important carbohydrates and act as carbon source to fulfill the energy requirements of plant cells during in vitro growth. Sucrose has an important function in AR induction and development through the instant availability of its hydrolytic products such as glucose and fructose in the culture media. The later monosaccharides can easily be transported to the plant cell mainly by active transport and partially by passive transport, where they influence root growth and development [40]. During optimization experiments, 1.0 mg/L NAA showed promising results in AR induction and growth on solid MS media; for the assessment of the differential effects of sucrose on AR induction and biomass formation, leaf explants were further cultured on MS media containing NAA (1.0 mg/L) and supplemented with different concentrations of sucrose (1, 2, 3, 4, 5 and 6%). The results indicated that 4% sucrose instigated the highest AR induction frequency (90 ± 4.25) and maximum AR biomass (fresh biomass: 17.7 g/L) in leaf explants within three days of culture cultivation, compared to other concentrations of sucrose tested (Table 2; Figure 1). When the concentration of sucrose exceeded or decreased 4%, the AR induction frequency and growth rate were significantly reduced, wherein 59% and 53% (AR induction frequency) and 11.2 g/L and 10.2 g/L (fresh biomass) were observed at 6% and 1% sucrose, respectively (Table 2). Similarly, sucrose at higher concentrations (7–10% *w*/*v*) resulted in a prominent declination in AR induction and biomass formation in leaf explants of *Gynura procumbens*. However, optimal AR growth (fresh biomass: 13.8 ± 1.60 g) was observed in the media augmented with 2% *w*/*v* sucrose [41]. In another study, 3% *w*/*v* sucrose induced the maximum root biomass (fresh weight: 121.71 g/L) in the adventitious roots of *Hypericum perforatum L* [42]. Sucrose and its hydrolytic products including glucose and fructose act as signal compounds during in vitro growth and might directly influence the plant cell physiology, growth and development, thus promote enhanced AR induction and proliferation.

### 3.3. Effects of AR Pretreatment With Varying Levels of Elicitors at Different Time Periods on AR Growth in Liquid Culture Media

Shake flasks cell culture using liquid media facilitate the efficient bioprocessing of plant tissues. The prospects of bioprocess technology include production of biomass and enhancement of medicinally active metabolites; those may not be found in the counterpart natural plants. Moreover this technology provides a suitable model system to study and understand the plant cell cycle, effects of plant growth regulators or elicitors on the growth, physiological responses and secondary metabolism of plant cell during in vitro growth [18,31,43]. In this study, AR of *A. amygdalina* grown over solid media containing 1.0 mg/L NAA and 3% sucrose were taken and were transiently elicited (pre-treatment) with different elicitors for different time periods; after that, approximately 2 g/L of AR pretreated with each elicitor were transferred into liquid MS media containing 1.0 mg/L NAA and 3% sucrose, following incubation on rotary shaker for a 30-day culture period.

**Figure 2:**
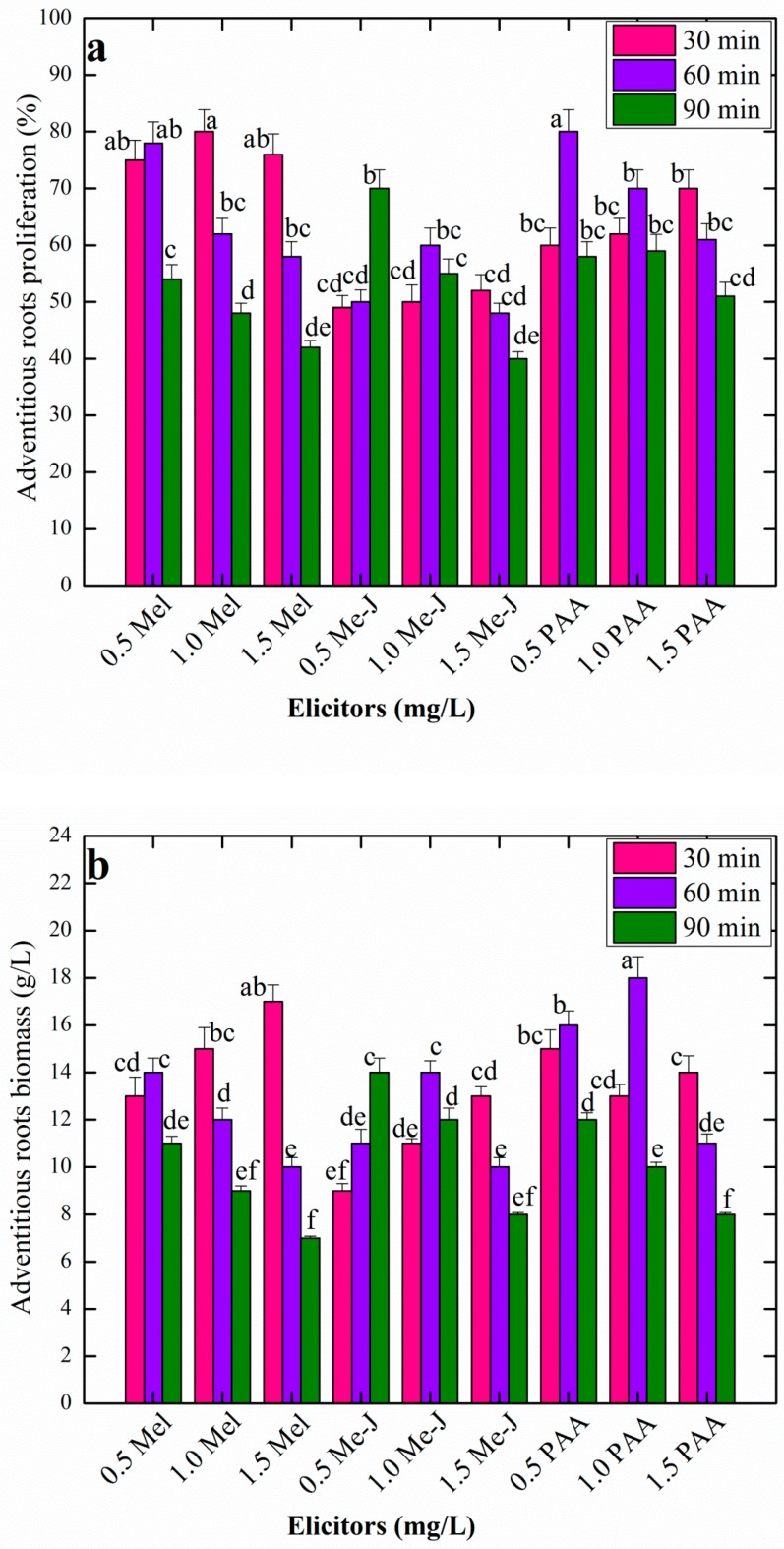
Effects of pretreatment of AR with different elicitors for different time periods on growth and biomass of AR in MS liquid media containing 1.0 mg/L NAA and 3% sucrose. (**a**) Percent AR morphogenetic response and (**b**) AR fresh biomass (g/L). Data represent the mean values of triplicates with ± standard error for each treatment in three repeated experiments. Annotation of columns data with different alphabet (s) represents the significance at (*P* = 0.05).

The data in Figure 2a,b indicate the AR growth in response to pretreatment with different elicitors for different time periods. The morphogenetic potential of AR (38% to 80%) was observed in response to all the elicitor treatments (Figure 2a). Among all the elicitor- treatments, melatonin (Mel: 1.0 mg/L), phenyl acetic acid (PAA: 0.5 mg/L) and methyl jasmonate (Me-J: 0.5 mg/L) resulted in higher AR growth of 80%, 60% and 80%, respectively, in shake flasks (Figure 2a). No study has previously reported on the impacts of chemical elicitors on the in vitro cell growth and development in *A. amygdalina;* however, all the elicitors employed in the current study have been well explored for their effects on growth in different plant cell culture studies in a variety of medicinal plants [21,31,44]. Uptake of methyl jasmonate by plant cell results in the mutilation of the plant cell outer layer; acting as a signal compound, it triggers the plant cell biochemical pathways through reprogramming common attributes of the plant cell, such as plasticity and totipotency. Consequently, activation of a cascade of unusual events takes place inside the plant cell and the plant cell gets induction and differentiation during in vitro growth [16,44]. As evidenced in this study, Me-J pretreatment has shown lower growth response in AR growth compared with PAA and Mel. Similarly lower growth response was observed in response to Me-J during AR suspension culture in *A. scoparia*; a medicinally important Artemisia plant species [32]. Contrarily, Me-J has positively influenced AR growth in context of AR induction and biomass formation in medicinally important plants, *A. bracteosa* and *S. rebaudiana* [31,44]. The growth-stimulating effects of Me-J and PAA as chemical elicitors in AR cultures have not been well explored. Rather, Me-J is reported for lower growth response in many medicinal plants to influence biomass formation. However, recently, both Me-J and PAA have been reported for their positive effects on induction and biomass accumulation of AR in a high valued medicinal plant *Ajuga bracteosa* [44]. Both Me-J and PAA can initiate a cascade of different metabolic events in plant cell as a result of the stress condition induced by these elicitors, consequently resulting in induction of AR and biomass formation in explants [21,31].

In context of time period and concentration of each elicitor tested, different responses in AR growth were observed, for instance, AR pretreatment with Mel at 0.5 mg/L for 60 min, at 1.0 mg/L for 30 min and at 1.5 mg/L for 30 min, which resulted in higher growth response and AR biomass formation (Figure 2a,b). Furthermore, PAA at 1.0 mg/L and 1.5 mg/L was more responsive when treated with explants for 60 min; however, at a higher dose (1.5 mg/L), it was more active for a 30 min time period. Interestingly, Me-J at a higher dose (1.5 mg/L) and lower dose (0.5 mg/L) was more active during AR growth when treated for 30 min and 90 min, respectively. Different studies have shown the positive effects of pre-treatment of explants with chemical elicitors for defined periods of time in induction and proliferation of AR. For instance, Kazmi et al. [31] reported direct AR induction frequencies of 88% and 86% in *Stevia rebaudiana*, when leaf explants were pre-treated with 0.5mg/L Me-J for 45 min and 1.0mg/L PAA for 15 min, respectively. Moreover, callus explants of *Fagonia indica* when treated with 0.5 mg/L Me-J and with 1.0 mg/L PAA for 2 h, maximum, resulted in higher AR induction frequencies of 88% and 80% respectively, in the cultures [21]. In this study, treatment of AR with higher levels of elicitors for maximum time period caused a reduction in AR response and proliferation in suspension cultures (Figure 2a,b). The adventitious rooting capacity in explants can be ceased at higher doses of elicitors, as they might inhibit AR formation and might promote callus organogenesis in explants [21,30,31]. Furthermore, at higher doses, the elicitors, especially Me-J, can inhibit growth and can cause cell necrosis and cell death if maintained for prolonged time during in vitro cultures [15,45].

Compared with data on AR biomass formation on solid media, no significant increase in biomass was observed in AR suspension cultures, established in response to pretreatment of AR with elicitors. On liquid media, maximum AR biomass (18 g/L) was observed at PAA (1.0 mg/L) treated cultures; this was followed by Mel, producing 17 g/L of fresh AR, when treated at 1.5 mg/L. Lower values in biomass formation were observed for Me-J treated cultures (Figure 2b). Liquid culture media has a profound impact on the bioprocessing of plant tissues; compared with solid media, rapid root growth and proliferation can be observed in plants grown in liquid media [46]. Melatonin, which is an animal hormone, produced good results in AR proliferation in this study. As the efficiency of melatonin on plant’s physiology is being studied, there are now several reports that give a sound perception about its role in plant cell division and root development [47], regulating circadian rhythms and other photoperiod dependent processes [31,48]. Moreover, it has been applied as a substitute to IAA because of structural similarities [49].

### 3.4. Effects of Elicitation on Antioxidant Profile of AR Raised in Vitro

**Figure 3:**
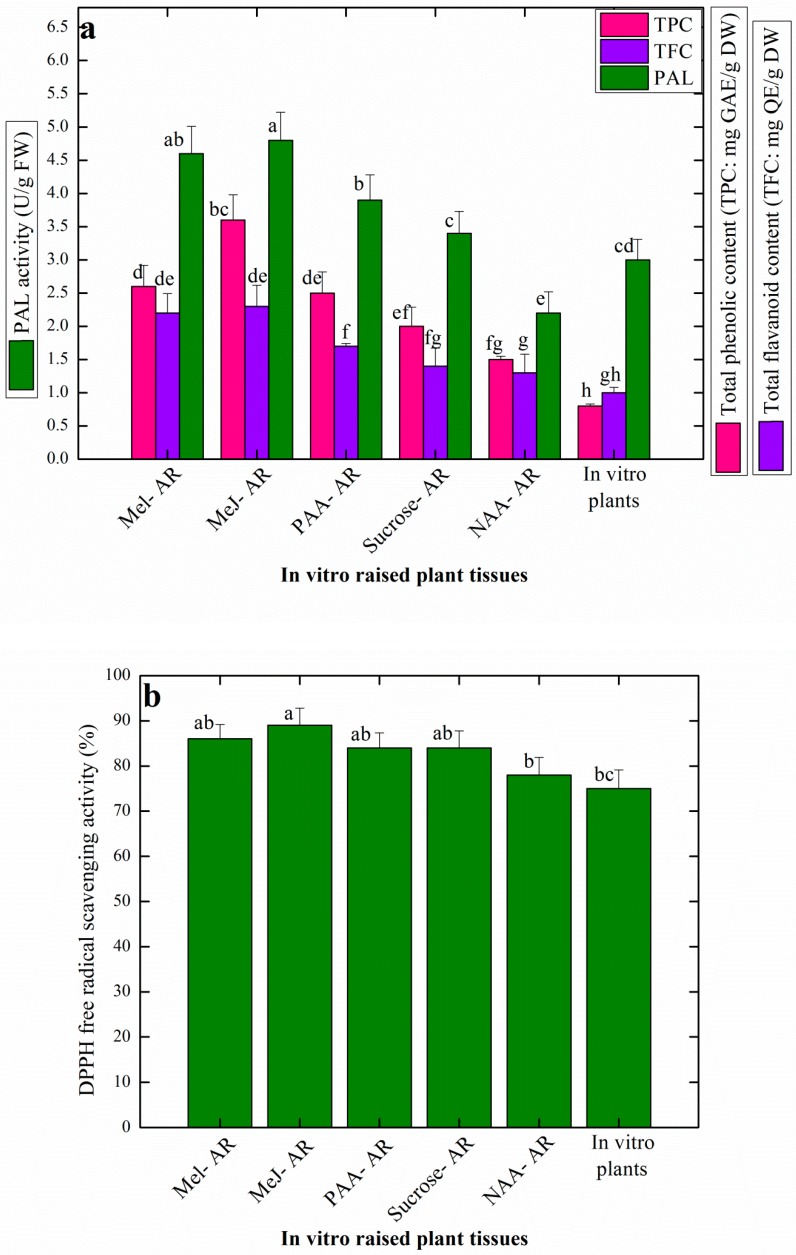
Evaluation of antioxidant secondary metabolites in the in vitro-raised AR in response to different elicitor treatments and control. (**a**) Total phenolic content (TPC), total flavonoid content (TFC) and phenylalanine ammonia-lyase activity; (**b**) DPPH free radical scavenging activity. Data represent the mean values of triplicates with ± standard error. Mel-AR: Melatonin treated AR, Me-J-AR: Methyl jasmonate treated AR, PAA-AR: Phenyl acetic acid treated AR, Sucrose-AR: AR established on solid MS media containing 1.0 mg/L NAA and 4% sucrose, NAA-AR: AR established on solid MS media containing 3% sucrose and 1.0 mg/L NAA. Data represent the mean values of triplicates with ± standard error. Annotation of columns data with different alphabet (s) represents the significance at (*P* = 0.05).

*Artemisia* species are well known to harbor sufficient amount of antioxidant metabolites including phenolic acids and flavonoids. The wild grown *A. amygdalina* has been profiled for the presence of phenolic and flavonoid compounds; however, production of these medicinally important secondary metabolites through plant in vitro cultures has not been well explored. In this study, the effects of elicitation on production of antioxidant potential in AR samples, harvested from the respective growth treatments, were evaluated through calorimetric tests. The results showed that AR pretreated with elicitors significantly enhanced the production of total phenolic content (TPC), total flavonoid content (TFC) and pheyl alanine ammonia lyase (PAL) activity in the AR grown in liquid media (Figure 3a). Methyl jasmonate (Me-J) treated AR resulted in the production of higher levels of TPC (3.6 mg), TFC (2.3 mg) and PAL (4.8 U/g × FW) (Figure 3a).

Similarly, Ali et al. [50] studied the effects of Me-J on the production of secondary metabolites in *Artemisia absinthium* suspension cultures. They concluded that in presence of 0.5 mg/L and 1.0 mg/L Me-J, cell cultured resulted in the maximum production of TPC (5.6 mg) and TFC (7.6 mg), respectively. No significant differences in the levels of TPC, TFC and PAL activity were observed in the Mel and PAA treated AR samples, respectively. However, AR grown over solid MS media in presence of 4% sucrose and 1.0 mg/L NAA, were found to accumulate higher levels of TPC (2 mg), TFC (1.4 mg) and PAL (3.8U/g × FW) when compared with AR established in the presence of 3% sucrose and 1.0 mg/L NAA on the same media, showing production of TPC (1.5 mg), TFC (1.3 mg) and PAL (3.4 U/g), respectively (Figure 3a). Sucrose at higher levels (4–7%) has shown positive effects on production of TPC and TFC in a related Artemisia plant: *A. absinthium*, wherein 5% sucrose supplementation in the media resulted in the highest total phenolic content (5.65 mg), while 7% sucrose was optimal for the enhanced production of total flavonoid content (1.45 mg), respectively [51]. It can be inferred that sucrose in the culture media may create a situation of an osmotic stress in the plant cells, and thus, influence plant cell growth and secondary metabolism, which results in the production of plant polyphenols [42].

Elicitation is one of the most successful strategies used in vitro for the enhancement of the production of plant secondary metabolites in a lot of medicinal plants, in order to meet the higher industry demands for making pharmaceutical products and drugs [2]. Similar to chemical elicitors such as Me-J, Mel and PAA, physical elicitors for instance continuous light illumination or dark condition during in vitro growth are also used routinely to enhance production of total phenolic and flavonoid compounds in plants species [38,52]. In one study, maximum production of TPC (8.2 mg/g DW) and TFC (2.5 mg/g DW) were recorded in cell cultures of *A. amygdalina* grown under continuous light and dark conditions, respectively [53]. In a recently published report, Me-J treatment resulted in production of higher amounts of TPC (2.3 mg) and TFC (3.9 mg) respectively, in AR cultures of *Stevia rebaudiana* [31].

In comparison to AR samples, lower values of TPC, TFC and PAL activity were observed in the in vitro raised plant (control sample). It is worth mentioning that liquid media significantly enhanced the production of these important secondary metabolites in AR than the AR established on solid media. The production of TPC and TFC, which basically represent the antioxidant compounds in a plant tissue, was found in correlation to the expression of PAL activity (Figure 3a). This direct correlation might be due to the overexpression of PAL isozymes; normally during biosynthesis of phenolic acids and flavonoids PAL, turnover occurs [31]. PAL has an important role in the phenyl propanoid pathway and its regulation is directly linked with biosynthesis of a variety of flavonoids, volatiles and phenolic compounds [16].

Interestingly, the antioxidant activity in the present study was observed at almost in a similar fashion in all the plant samples (Figure 3b). All the elicitor treatments resulted in higher antioxidant potential in the AR, wherein the Me-J treated AR cultured produced the highest free radical scavenging activity of 89% (Figure 3b). Sucrose-induced AR and PAA induced AR resulted in a similar antioxidant activity (84%). In a similar study, highest antioxidant activity (87%) was recorded in the Me-J treated AR suspension cultures of *Artemisia scoparia* [32]. In comparison with other testing methods for evaluation of antioxidant potential, DPPH^o^ free radical scavenging assay is preferred for its optimal performance and authentic results. Nonetheless, it is a robust, simple, easy and reliable method to test the antioxidant potential of a plant tissue [16]. Antioxidant activity in a plant cell is directly linked with the production of distinctive low mass compounds, also called secondary metabolites because of their less involvement in the primary metabolism [30]. These compounds represent different classes, for instance, phenolic acids, alkaloids, tannins, flavonoids, organic volatiles etc. Altogether, these secondary metabolites are produced in a plant cell as a result of some surrounding stress stimuli [15]. The byproducts of the intense stress stimuli in the form of singlet oxygen or nitrogen known as reactive oxygen species (ROS) act as inhibitors of cell membrane and DNA, and may cause necrosis or cell death. In order to survive normal growth and development, plant cells mitigate the effects of these devastative ROS through activation of its enzymatic and proteinous antioxidant systems [38]. Upon activation of the antioxidant system, plant cells produce secondary metabolites, which come in contact with the ROS and scavenge them, and thus, prevent the hazards created by them. Eventually, plant cells attain their normal route of growth and development. However, these secondary metabolites, such as phenolic acids, flavonoids, terpenes and alkaloids etc, are of anthropogenic interest as they are in high demand in pharmaceutical industries for the preparation of a variety of medicines [17]. Generally, elicitors acting as stress inducers are more potent to induce and stimulate the antioxidant potential in plant cells [50]. In support of our data, the role of Me-J on induction of secondary metabolism and production of antioxidants in plants is well reported in the literature and it is believed that this elicitor acts to reprogram cell metabolism through activation of transcription factors and cell cycle progression [21,31].

### 3.5. Effects of Elicitation on Essential Oil Profiles of AR Raised in Vitro

**Figure 4:**
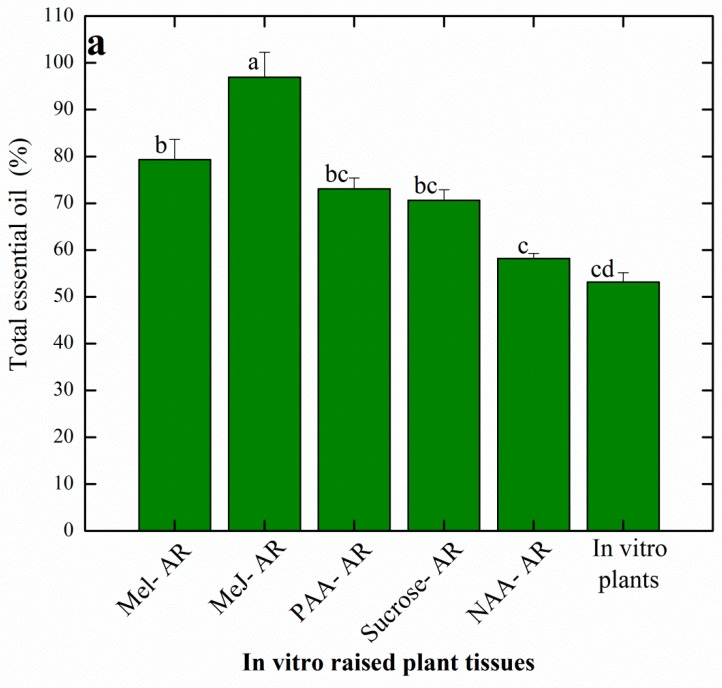

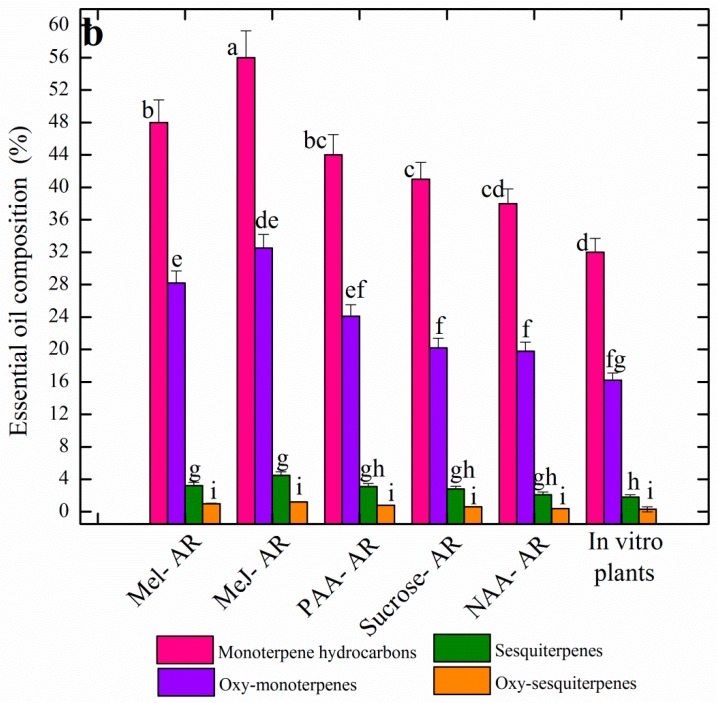
Evaluation of essential oil biosynthesis in the in vitro raised AR in response to different elicitor treatments and control. (**a**) Total essential oil (%); (**b)** essential oil composition (%). Mel-AR: Melatonin treated AR, Me-J-AR: Methyl jasmonate treated AR, PAA-AR: Phenyl acetic acid treated AR, Sucrose-AR: AR established on solid MS media containing 1.0 mg/L NAA and 4% sucrose, NAA-AR: AR established on solid MS media containing 3% sucrose and 1.0 mg/L NAA. Data represent the mean values of triplicates with ± standard error. Annotation of columns data with different alphabet (s) represents the significance at (*P* = 0.05).

In the present study, pre-treatment with different elicitors resulted in the enhanced accumulation of essential oils in AR culture of *A. amygdalina*. Among all the selected samples, maximum total essential oil accumulation (96%) was observed in Me-J treated AR followed by Mel (79%) and PAA (73%), respectively. However, a moderate level (70%) was observed in the sucrose-induced AR and the lowest total essential oil (53%) was recorded for the in vitro raised plant (Figure 4a). Essential oil profiles of the different AR cultures presented accumulation of monoterpene hydrocarbon (32–56%), oxygenated monoterpens (16–32%), sesquiterpenes (1.8–4.5%) and oxygenated sesquiterpene (0.3–1.2%) in response to the different elicitor treatments (Figure 4b). Overall, higher levels of these different classes of terpene volatiles were observed in Me-J treated AR and lower values were observed in the in vitro raised plant.

A substantial number of volatile compounds were detected by GC-MS analysis in the present study. However, those compounds were selected that were detected at a considerable level in all of the six selected plant cell lines. In total, 20 compounds belonging to various terpene classes were quantified and compared among the selected samples. Results revealed biosynthesis of the major bioactive compounds such as α-thujene (6.8%), α-pinene (8.3%), 1,8-cineole (16.2%), camphor (8.4%) and verbenole (10.2%) in the Me-J treated AR. Accordingly, methyl jasmonate (Me-J) has been reported for its influential role in the maximum accumulation of terpene volatiles in cell suspension cultures of *Ajuga bracreosa* and *Achillea gypsicola*, respectively [54,55]. Contrarily, lower values of essential oils were observed in shoot cultures of *Sautreja khuzistanica* in response to Me-J [56].

Compared with other cell lines, a higher level of α-terpineol (6.7%) was detected in the PAA treated AR. Furthermore, the maximum production of pinocarvone (8.9%) was recorded in the AR grown over solid media in presence of NAA (Table 3). Sucrose-mediated AR resulted in higher production of piperitone (10.6%) compared to other plant samples. Nonetheless, the in vitro plants (control sample) showed production of higher levels of L-borneol (9.25%) and β-pinene (12.8%), respectively. Generally, terpene biosynthesis in the plant cell starts with the metabolic regulation of two important precursors known as isopentenyl pyrophosphate (IPP) and dimethylallyl diphosphate (DMAPP). It is worth mentioning that IPP in higher plants is biosynthesized through two different metabolic pathways, including the mevalonatedependent (MVA) pathway and mevalonate-independent pathway, also known as the deoxyxylulose phosphate pathway [30,54]. The higher production of the terpene volatiles in AR cultures in the present study can be collaborated with the modulatory effects of chemical elicitors such as Me-J in the biosynthesis of essential oils. The process of elicitation is directly linked with the biosynthesis of essential oils in the plant cell through its upregulation of the biosynthetic pathways. Several factors are responsible for the regulation of volatiles biosynthesis. These factors include the genetic makeup of the explant used in cell cultures, type of culture media, ontology of the growing cells and the in vitro developmental phase [2,15]. The different volatiles compounds detected and quantified in the current study are known for their diverse biological activities. The monoterpenes are of particular interest in many industrial applications, including food, fragrance, cosmetic and pharmaceuticals. In the food industry, the monoterpenes are attributed to their diverse applications as natural organic solvents with an eco-friendly trademark and prominent diversity. Large varieties of monoterpene organic solvents obtained from essential oils can efficiently replace the use of conventional solvents such as petroleum or halogenated solvents in a variety of extraction procedures [30]. Amongthe different potent terpenes as produced in higher quantities in this study, 1,8-cineole is widely known for its application in pharmaceuticals products as a strong anti-microbial, anti-inflammatory and gastroprotective agent [57]. Camphor is a vital component of Ayurvedic system of different countries such as China and Japan, where camphor is used as an anesthetic agent. It is also used for fragrance in cosmetics, as food additive for taste improvement and as preservative in bakery products [58]. α-pinene has been used widely as a food flavoring additive with no reported harms on human health as approved by the FDA. Moreover, it is a potent antimicrobial, hypertensive, anti-inflammatory and anticeptive agent [59]. Piperitone, borneol, pinene, terpinene and pinocarvone are other important terpenes produced in AR cultures of *A. amygdalina*, which are important volatiles used in different biological and pharmacological applications including antifungal, antibacterial, anti-inflammatory, antioxidant, anti-stress, anticeptive and analgesic activities [60,61,62]. Besides their medicinal applications, these volatile compounds are widely used in the cosmetics industry as fragrance components and in the food industry to improve the aroma of different types of foods [30].

## 4. Conclusions

For the first time, Adventitious roots (AR) were established in *A. amygdalina*; a critically endangered medicinal plant. On solid MS media containing 1.0 mg/L NAA and 4% sucrose, higher AR induction frequency and growth were observed in leaf explants. Transient elicitation of AR followed by growth in liquid media resulted in the enhanced production of phenolic compounds and essential oil. Among the different elicitors, Me-J showed modulating effects on plant cell secondary metabolism and biosynthesis of terpenes. The present study suggests a suitable method for the production of industrially important essential oils. It further provides a sustainable method for production of AR and also provides a promising ex-situ conservation method for the protection of this critically endangered plant *A. amygdalina*. Genomic and transcriptomic studies are further recommended to enhance the essential oils production through AR cultures in *A. amygdalina.*

## Figures and Tables

**Table 1:** Differential response of adventitious rooting with varying concentrations and types of auxins growing on solid MS medium with 3% sucrose after four weeks of incubation.

Auxins	Concentration (mg/L)	Percent Root Induction	Number of Days to Root Initiation	Mean Root Number	Mean root Length (cm)	Fresh Biomass (g/L)
Control	0.0	0.0	0.0	0.0	0.0	
IBA	0.5	62 ± 2.67 ^d^	5	2.6 ± 0.39 ^c^	1.15 ± 0.11 ^cd^	9.5 ± 2.6 ^b^
	1.0	76 ± 3.78 ^b^	5	4.1 ± 0.45 ^ab^	2.3 ± 0.12 ^bc^	12.3 ± 3.1 ^ab^
	1.5	67 ± 2.83 ^c^	5	1.7 ± 0.3 ^cd^	1.12 ± 0.13 ^c^	9.2 ± 2.7 ^b^
NAA	0.5	78 ± 3.84 ^ab^	4	3.8 ± 0.43 ^b^	2.06 ± 0.05 ^b^	12.3 ± 3.1 ^ab^
	1.0	87 ± 4.25 ^a^	4	6.8 ± 0.48 ^a^	3.2 ± 0.08 ^a^	15.7 ± 3.5 ^a^
	1.5	71 ± 3.72 ^bc^	4	2.9 ± 0.45 ^bc^	1.3 ± 0.08 ^cd^	10.2 ± 2.8 ^bc^
IAA	0.5	–	–	–	–	–
	1.0	–	–	–	–	–
	1.5	–	–	–	–	–

Data represent the mean values of triplicates with ± standard error for each treatment in three repeated experiments. Means sharing the same letter are not significantly different (*P* = 0.05). IBA: Indole-3-butyric acid; NAA: α-naphthalene acetic acid. IAA: Indole-3- acetic acid; ‘0.0’ or ‘-’: no result.

**Table 2:** Sucrose-induced adventitious root growth in optimized cultures after four weeks of incubation.

Sucrose (*w*/*v*)	Percent Root Induction	Number of Days to Root Initiation	Mean Root Number	Mean Root Length (cm)	Fresh Biomass (g/L)
**0**	0.0	0.0	0.0	0.0	
**1**	53 ± 2.67 ^c^	5	3.6 ± 0.39 ^d^	2.15 ± 0.11 ^c^	10.5 ± 2.6 ^c^
**2**	65 ± 3.78 ^bc^	5	4.8 ± 0.45 ^c^	2.80 ± 0.12 ^bc^	12.3 ± 3.1 ^bc^
**3**	87 ± 4.25 ^ab^	4	6.8 ± 0.48 ^b^	3.2 ± 0.08 ^b^	15.7 ± 3.5 ^ab^
**4**	90 ± 4.25 ^a^	3	7.8 ± 0.48 ^a^	4.20 ± 0.08 ^a^	17.7 ± 3.5 ^a^
**5**	71 ± 3.72 ^b^	3	4.1 ± 0.43 ^c^	2.06 ± 0.05 ^c^	13.3 ± 3.1 ^b^
**6**	59 ± 2.67 ^c^	4	3.9 ± 0.45 ^cd^	1.80 ± 0.08 ^d^	11.2 ± 2.8 ^c^

Data represent the mean values of triplicates with ± standard error for each treatment in three repeated experiments. Means sharing the same letter are not significantly different (*P* = 0.05).

**Table 3:** Composition of the Essential oils in different in vitro raised AR samples of *A. amygdalina*.

S.no	Compound	Class	Molecular Formula	Mel-AR (%)	Me-J-AR (%)	PAA-AR (%)	Sucrose-AR (%)	NAA-AR (%)	In vitro Plants
1	α-Thujene	Monoterpene hydrocarbons	C_10_H_16_	4.71	6.8	4.2	3.2	3	2.5
2	α-Pinene	Monoterpene hydrocarbons	C_10_H_16_	7.2	8.3	6.3	5.2	4.8	2.8
3	δ-Camphene	Monoterpene hydrocarbons	C_10_H_16_	4.4	5.5	4.2	4.0	3.8	3
4	Camphene	Monoterpene hydrocarbons	C_10_H_16_	5.12	6.4	4.1	3.5	3.2	2.5
5	β-Pinen	Monoterpene hydrocarbons	C_10_H_16_	4.21	6.5	3.2	2.8	2.5	12.8
6	α–phellandrene	Monoterpene hydrocarbons	C_10_H_16_	5.15	6.1	4.2	3.8	3.6	3
7	1,8-Cineole	Oxygenated monoterpenes	C_10_H_18_O	12.4	16.2	8.14	5.36	4.8	4.2
8	γ-Terpinene	Monoterpene hydrocarbons	C_10_H_16_	5.37	6.2	4.2	3.4	3	2.4
9	Camphor	Oxygenated monoterpenes	C_10_H_16_	7.1	8.4	5.4	4.5	3.5	1.0
10	L-Borneol	Oxygenated monoterpenes	C_10_H_18_O	6.5	trace	9.0	6.8	6.1	9.25
11	α-Terpineol	Monoterpene hydrocarbons	C_10_H_18_O	3.84	2.9	6.7	5.4	trace	5.23
12	β-Elemene	Sesquiterpene hydrocarbons	C_15_H_24_	-	4.5	-	0.85	1.1	0.5
13	Piperitone	Monoterpene hydrocarbons	C_10_H_16_O	trace	2.1	1.9	10.6	8.5	2
14	Valencene	Oxygenated sesquiterpnes	C_15_H_24_	trace	trace	trace	trace	0.4	0.3
15	Verbenol	Oxygenated monoterpenes	C_10_H_16_O	5.1	10.2	4.2	3.5	trace	1.2
16	Pinocarvone	Oxygenated monoterpenes	C_10_H_14_O	4.2	5.6	3.1	4.54	8.9	1.55
17	β-bisabolene	Sesquiterpene hydrocarbons	C_15_H_24_	3.0	-	-	0.75	1.0	0.8
18	Nerolidol	Sesquiterpene hydrocarbons	C_15_H_26_O	trace		3.1	1.2	-	0.5
19	α-farnesene	Sesquiterpene hydrocarbons	C_15_H_24_	1.0	1.2	1.1	0.6	trace	-
20	Caryophyllene oxide	Oxygenated sesquiterpnes	C_15_H_24_O	trace	-	trace	trace	trace	trace
